# RETRACTED: Inchingolo et al. Precision Medicine on the Effects of Microbiota on Head–Neck Diseases and Biomarkers Diagnosis. *J. Pers. Med.* 2023, *13*, 933

**DOI:** 10.3390/jpm16110562

**Published:** 2026-10-30

**Authors:** Angelo Michele Inchingolo, Giuseppina Malcangi, Fabio Piras, Giulia Palmieri, Vito Settanni, Lilla Riccaldo, Roberta Morolla, Silvio Buongiorno, Elisabetta de Ruvo, Alessio Danilo Inchingolo, Antonio Mancini, Francesco Inchingolo, Gianna Dipalma, Stefania Benagiano, Gianluca Martino Tartaglia, Assunta Patano

**Affiliations:** 1 Department of Interdisciplinary Medicine, University of Bari “Aldo Moro”, 70121 Bari, Italy; angeloinchingolo@gmail.com (A.M.I.); giuseppinamalcangi@libero.it (G.M.); dott.fabio.piras@gmail.com (F.P.); giuliapalmieri13@gmail.com (G.P.); v.settanni@libero.it (V.S.); l.riccaldo@studenti.uniba.it (L.R.); robertamorolla@gmail.com (R.M.); silvio.buongiorno@gmail.com (S.B.); studio.deruvo@libero.it (E.d.R.); ad.inchingolo@libero.it (A.D.I.); dr.antonio.mancini@gmail.com (A.M.); francesco.inchingolo@uniba.it (F.I.); studiobenagiano@gmail.com (S.B.); assuntapatano@gmail.com (A.P.); 2 Department of Biomedical, Surgical and Dental Sciences, School of Dentistry, University of Milan, 20122 Milan, Italy; gianluca.tartaglia@unimi.it; 3 UOC Maxillo-Facial Surgery and Dentistry, Fondazione IRCCS Ca Granda, Ospedale Maggiore Policlinico, 20122 Milan, Italy

The journal retracts the article titled “Precision Medicine on the Effects of Microbiota on Head–Neck Diseases and Biomarkers Diagnosis” [1], cited above.

Following publication, concerns were brought to the attention of the Editorial Office relating to the accuracy and relevancy of several citations in this publication [1].

In accordance with the journal’s standard procedures, the Editorial Office and the Editorial Board conducted an investigation that confirmed the presence of several citations that lacked sufficient relevance to the article’s content. The authors collaborated with the investigation and proposed revisions. However, the Editorial Board concluded that the proposed correction would not fully address the concerns identified. 

As a result, the Editorial Board has decided to retract the article in accordance with MDPI’s retraction policy.

This retraction was approved by the Editor-in-Chief of the *Journal of Personalized Medicine*.

The authors have been informed of this retraction but did not provide a comment on this decision.

## References

[1] Inchingolo A.M., Malcangi G., Piras F., Palmieri G., Settanni V., Riccaldo L., Morolla R., Buongiorno S., de Ruvo E., Inchingolo A.D. (2023). RETRACTED: Precision Medicine on the Effects of Microbiota on Head–Neck Diseases and Biomarkers Diagnosis. J. Pers. Med..

